# Supplementary material for: Light-dependent phosphorylation of THRUMIN1 regulates its association with actin filaments and 14-3-3 proteins
Source: Plant Physiol. 2021 Aug 6;187(3):1445–61. doi: 10.1093/plphys/kiab374 (PMC8566215; doi:10.1093/plphys/kiab374)
Supplement: kiab374_Supplementary_Data [file kiab374_supplementary_data.zip › PP2021RA00446R1_Supplemental_Figures S1S3.pdf]

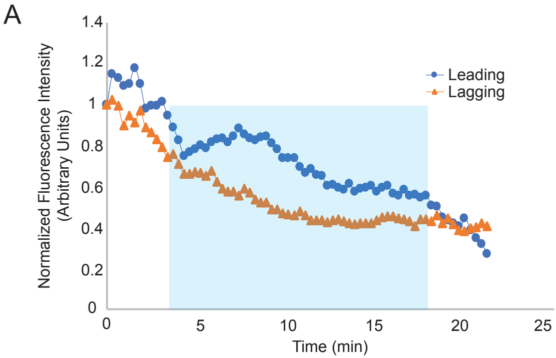

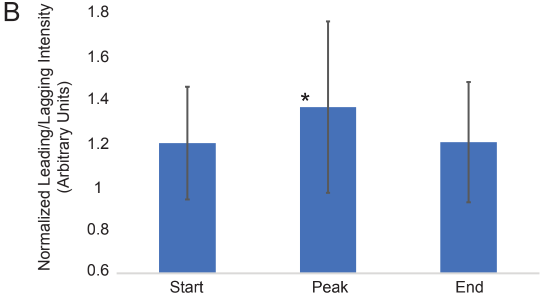

Supplemental Figure S1. THRUMIN1-cp-actin localization increased at the leading edge of Arabidopsis palisade mesophyll chloroplasts in response to blue light. (A) Representative time-course plot of fluorescence intensities of THRUMIN1:YFP at the leading edge of a chloroplast versus the lagging edge of the chloroplast measured with KymographBuilder (see methods). Upon exposure to a high blue light microbeam (at 4 minutes, blue highlighting), the fluorescence intensity of both the leading and lagging edge initially decreased, which was followed by an increase in THRUMIN1:YFP fluorescence intensity at the leading edge while the fluorescence intensity continued to decrease along the lagging edge. As the time course progressed, the leading edge fluorescence intensities of THRUMIN1:YFP decreased as the chloroplast moved out of the blue light microbeam. (B) Ratios of leading to lagging edge fluorescence intensities 2 minutes before the fluorescence at the leading edge reached its peak, at its peak, and 2 minutes after the peak. The data were collected from 13 chloroplasts from 7 cells. A paired two-tail T-test showed that the leading/lagging-edge fluorescence ratio is statistically significant (*p=0.0291) at the peak of movement. Error bars = standard deviation.

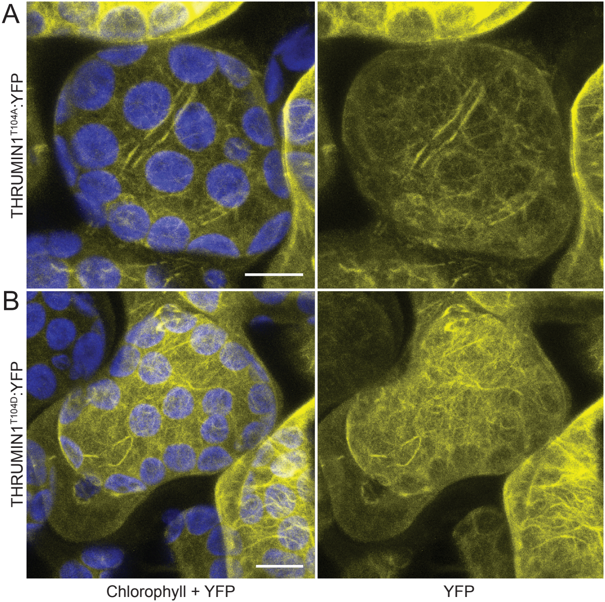

Supplemental Figure S2. Threonine 104 does not alter the filamentous localization of THRUMIN1. (A) *N. benthamiana* cells transiently expressing 35S:THRUMIN1^{T104A}:YFP displayed no alterations to the localization pattern of wild type THRUMIN1. (B) Similarly, the phosphomimetic variant 35S:THRUMIN1^{T104D}:Y-FP did not alter the localization of THRUMIN1 when expressed transiently in *N. benthamiana*. A and B, Representative time-lapse images are shown (514nm for YFP excitation) and chlorophyll autofluorescence is false-colored blue while the YFP channel is false-colored yellow. The scale bars indicate a 5 µm distance. Similar observations were made in at least 3 independent time-lapse movie replicates for each experiment.

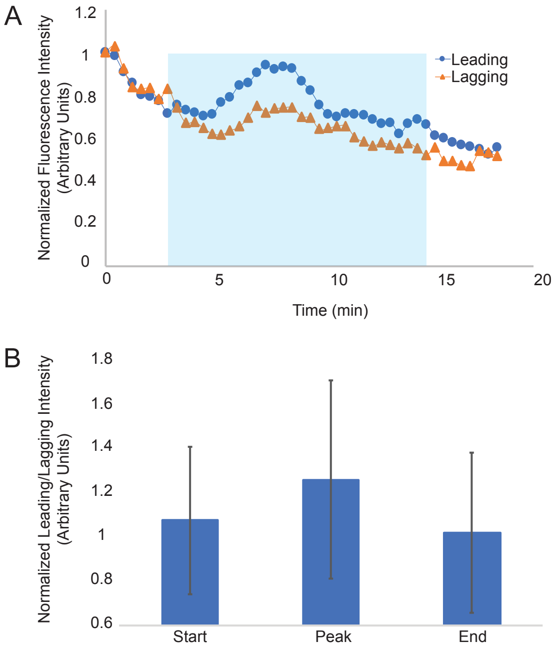

Supplemental Figure S3. Mutations to THRUMIN1's conserved cysteines altered reorganization of the cp-actin at the chloroplast periphery. (A) Representative time-course plot of fluorescence intensities of leading and lagging edges of mesophyll chloroplasts in Arabidopsis expressing 35S:THRUMIN1$^{C317/320/351/354A}$:YFP as measured with KymographBuilder (see methods). Upon exposure to a high blue light microbeam (at 2.6 minutes, blue highlighting), the fluorescence intensity of both the leading and lagging edge decreased followed by an increase in both the leading and lagging edge fluorescence intensity. As the time course progressed, the leading and lagging edge fluorescence intensities of THRUMIN1$^{C317/320/351/354A}$:YFP fluctuated and there was very little chloroplast movement as seen in Fig. 9. (B) Ratios of leading to lagging edge fluorescence intensities 2 minutes before the fluorescence at the leading edge reached its peak, at its peak, and 2 minutes after the peak. The data were collected from 15 chloroplasts from 7 cells. The results show that the leading/lagging-edge fluorescence ratio was not statistically significant (p=.0575, paired two-tailed T-test) with the 35S:THRUMIN1$^{C317/320/351/354A}$:YFP mutant. Error bars = standard deviation.
